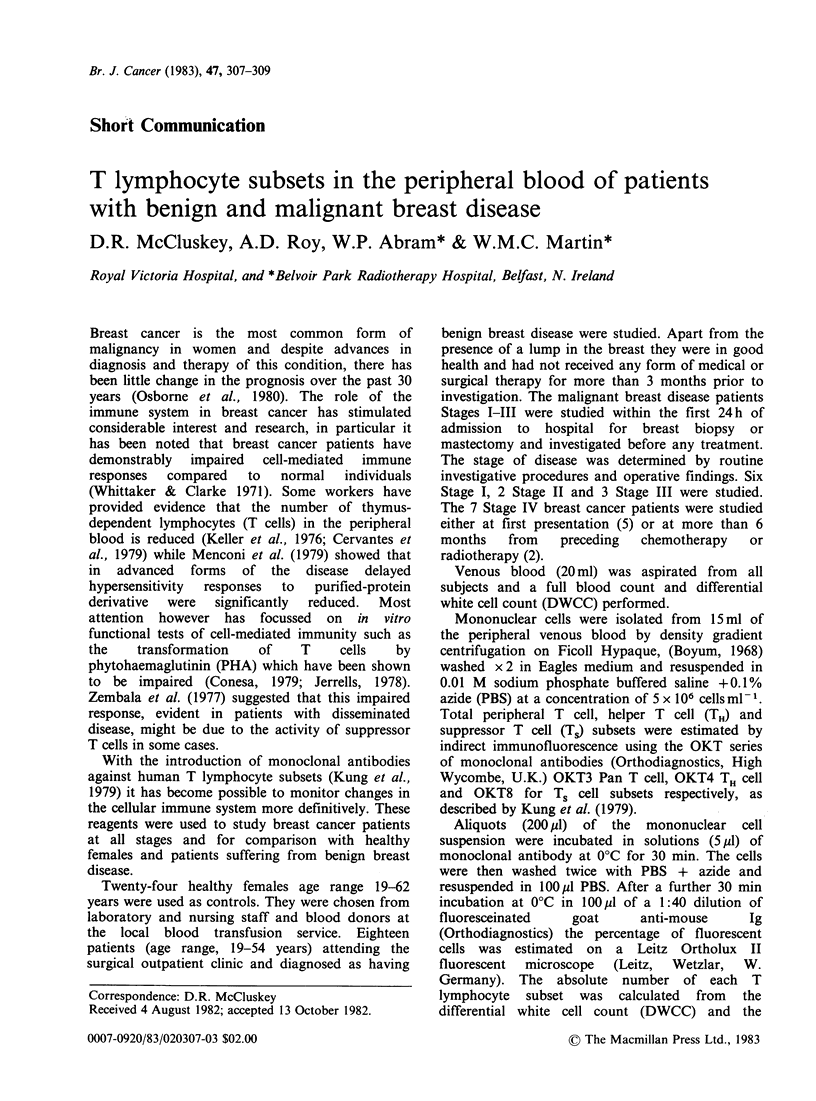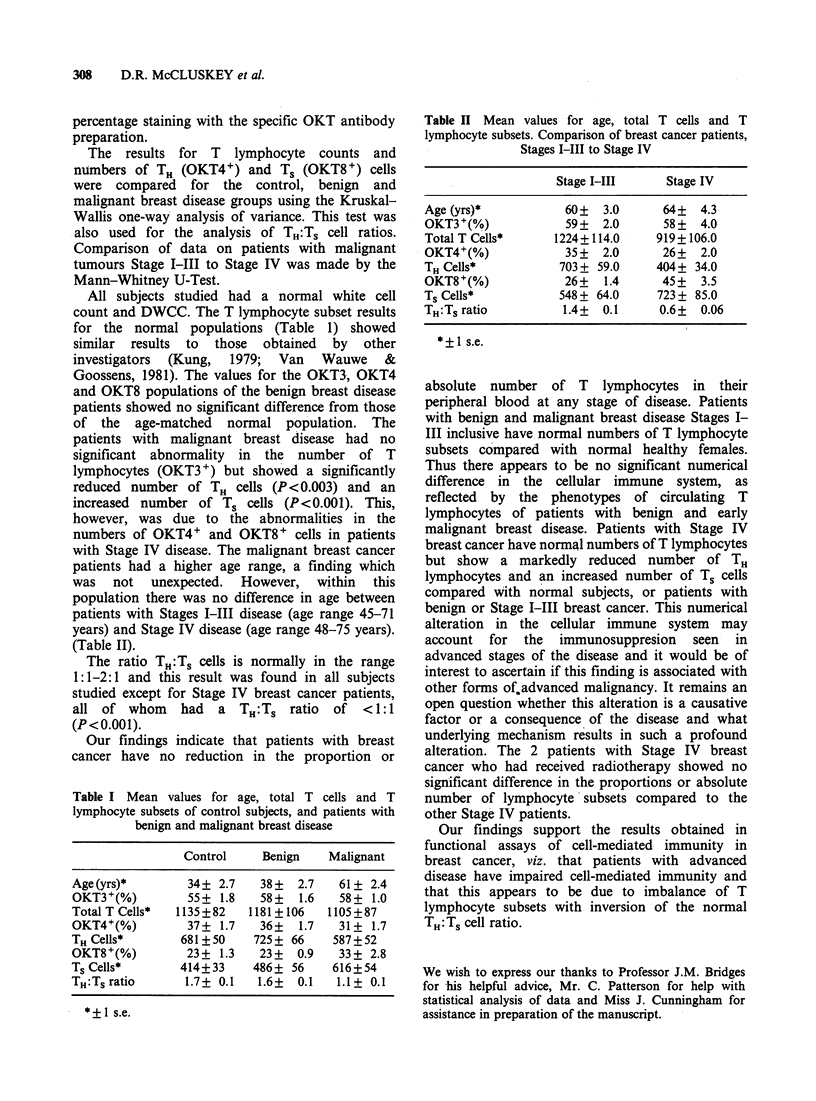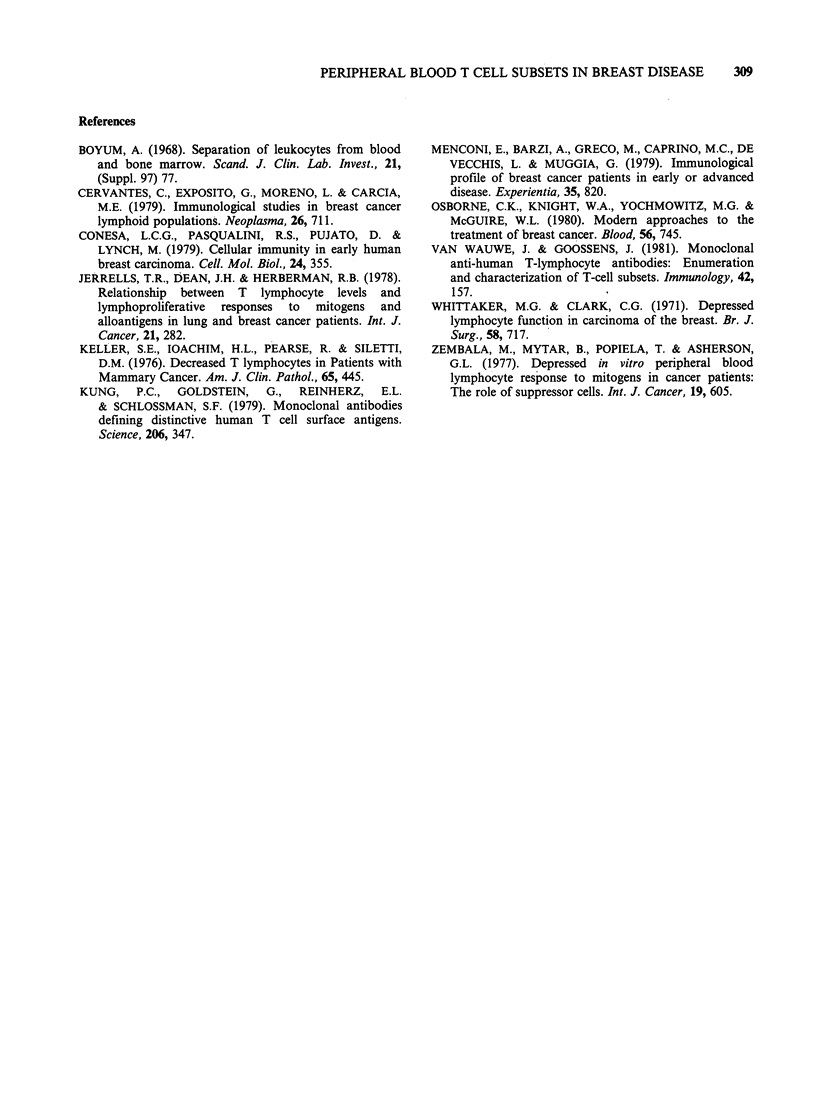# T lymphocyte subsets in the peripheral blood of patients with benign and malignant breast disease.

**DOI:** 10.1038/bjc.1983.41

**Published:** 1983-02

**Authors:** D. R. McCluskey, A. D. Roy, W. P. Abram, W. M. Martin


Br. J. Cancer (1983), 47, 307–309

**Short Communication**

### T lymphocyte subsets in the peripheral blood of patients with benign and malignant breast disease

D.R. McCluskey, A.D. Roy, W.P. Abram* & W.M.C. Martin*

*Royal Victoria Hospital, and *Belvoir Park Radiotherapy Hospital, Belfast, N. Ireland*

Breast cancer is the most common form of malignancy in women and despite advances in diagnosis and therapy of this condition, there has been little change in the prognosis over the past 30 years (Osborne *et al.*, 1980). The role of the immune system in breast cancer has stimulated considerable interest and research, in particular it has been noted that breast cancer patients have demonstrably impaired cell-mediated immune responses compared to normal individuals (Whittaker & Clarke 1971). Some workers have provided evidence that the number of thymus-dependent lymphocytes (T cells) in the peripheral blood is reduced (Keller *et al.*, 1976; Cervantes *et al.*, 1979) while Menconi *et al.* (1979) showed that in advanced forms of the disease delayed hypersensitivity responses to purified-protein derivative were significantly reduced. Most attention however has focussed on *in vitro* functional tests of cell-mediated immunity such as the transformation of T cells by phytohaemaglutinin (PHA) which have been shown to be impaired (Conesa, 1979; Jerrells, 1978). Zembala *et al.* (1977) suggested that this impaired response, evident in patients with disseminated disease, might be due to the activity of suppressor T cells in some cases.

With the introduction of monoclonal antibodies against human T lymphocyte subsets (Kung *et al.*, 1979) it has become possible to monitor changes in the cellular immune system more definitively. These reagents were used to study breast cancer patients at all stages and for comparison with healthy females and patients suffering from benign breast disease.

Twenty-four healthy females age range 19–62 years were used as controls. They were chosen from laboratory and nursing staff and blood donors at the local blood transfusion service. Eighteen patients (age range, 19–54 years) attending the surgical outpatient clinic and diagnosed as having benign breast disease were studied. Apart from the presence of a lump in the breast they were in good health and had not received any form of medical or surgical therapy for more than 3 months prior to investigation. The malignant breast disease patients Stages I–III were studied within the first 24 h of admission to hospital for breast biopsy or mastectomy and investigated before any treatment. The stage of disease was determined by routine investigative procedures and operative findings. Six Stage I, 2 Stage II and 3 Stage III were studied. The 7 Stage IV breast cancer patients were studied either at first presentation (5) or at more than 6 months from preceding chemotherapy or radiotherapy (2).

Venous blood (20 ml) was aspirated from all subjects and a full blood count and differential white cell count (DWCC) performed.

Mononuclear cells were isolated from 15 ml of the peripheral venous blood by density gradient centrifugation on Ficoll Hypaque, (Boyum, 1968) washed ×2 in Eagles medium and resuspended in 0.01 M sodium phosphate buffered saline +0.1% azide (PBS) at a concentration of $5\times10^{6}$ cells ml$^{-1}$. Total peripheral T cell, helper T cell ($T_H$) and suppressor T cell ($T_S$) subsets were estimated by indirect immunofluorescence using the OKT series of monoclonal antibodies (Orthodiagnostics, High Wycombe, U.K.) OKT3 Pan T cell, OKT4 $T_H$ cell and OKT8 for $T_S$ cell subsets respectively, as described by Kung *et al.* (1979).

Aliquots (200 $\mu$l) of the mononuclear cell suspension were incubated in solutions (5 $\mu$l) of monoclonal antibody at 0°C for 30 min. The cells were then washed twice with PBS + azide and resuspended in 100 $\mu$l PBS. After a further 30 min incubation at 0°C in 100 $\mu$l of a 1:40 dilution of fluoresceinated goat anti-mouse Ig (Orthodiagnostics) the percentage of fluorescent cells was estimated on a Leitz Ortholux II fluorescent microscope (Leitz, Wetzlar, W. Germany). The absolute number of each T lymphocyte subset was calculated from the differential white cell count (DWCC) and the

Correspondence: D.R. McCluskey
Received 4 August 1982; accepted 13 October 1982.

0007-0920/83/020307-03 $02.00

© The Macmillan Press Ltd., 1983

percentage staining with the specific OKT antibody preparation.

The results for T lymphocyte counts and numbers of $T_H$ ($OKT4^+$) and $T_S$ ($OKT8^+$) cells were compared for the control, benign and malignant breast disease groups using the Kruskal–Wallis one-way analysis of variance. This test was also used for the analysis of $T_H$:$T_S$ cell ratios. Comparison of data on patients with malignant tumours Stage I–III to Stage IV was made by the Mann–Whitney U-Test.

All subjects studied had a normal white cell count and DWCC. The T lymphocyte subset results for the normal populations (Table 1) showed similar results to those obtained by other investigators (Kung, 1979; Van Wauwe & Goossens, 1981). The values for the OKT3, OKT4 and OKT8 populations of the benign breast disease patients showed no significant difference from those of the age-matched normal population. The patients with malignant breast disease had no significant abnormality in the number of T lymphocytes ($OKT3^+$) but showed a significantly reduced number of $T_H$ cells ($P<0.003$) and an increased number of $T_S$ cells ($P<0.001$). This, however, was due to the abnormalities in the numbers of $OKT4^+$ and $OKT8^+$ cells in patients with Stage IV disease. The malignant breast cancer patients had a higher age range, a finding which was not unexpected. However, within this population there was no difference in age between patients with Stages I–III disease (age range 45–71 years) and Stage IV disease (age range 48–75 years). (Table II).

The ratio $T_H$:$T_S$ cells is normally in the range 1:1–2:1 and this result was found in all subjects studied except for Stage IV breast cancer patients, all of whom had a $T_H$:$T_S$ ratio of <1:1 ($P<0.001$).

Our findings indicate that patients with breast cancer have no reduction in the proportion or absolute number of T lymphocytes in their peripheral blood at any stage of disease. Patients with benign and malignant breast disease Stages I–III inclusive have normal numbers of T lymphocyte subsets compared with normal healthy females. Thus there appears to be no significant numerical difference in the cellular immune system, as reflected by the phenotypes of circulating T lymphocytes of patients with benign and early malignant breast disease. Patients with Stage IV breast cancer have normal numbers of T lymphocytes but show a markedly reduced number of $T_H$ lymphocytes and an increased number of $T_S$ cells compared with normal subjects, or patients with benign or Stage I–III breast cancer. This numerical alteration in the cellular immune system may account for the immunosuppresion seen in advanced stages of the disease and it would be of interest to ascertain if this finding is associated with other forms of advanced malignancy. It remains an open question whether this alteration is a causative factor or a consequence of the disease and what underlying mechanism results in such a profound alteration. The 2 patients with Stage IV breast cancer who had received radiotherapy showed no significant difference in the proportions or absolute number of lymphocyte subsets compared to the other Stage IV patients.

Our findings support the results obtained in functional assays of cell-mediated immunity in breast cancer, *viz.* that patients with advanced disease have impaired cell-mediated immunity and that this appears to be due to imbalance of T lymphocyte subsets with inversion of the normal $T_H$:$T_S$ cell ratio.

**Table I** Mean values for age, total T cells and T lymphocyte subsets of control subjects, and patients with benign and malignant breast disease

| | Control | Benign | Malignant |
|---|---|---|---|
| Age (yrs)* | 34± 2.7 | 38± 2.7 | 61± 2.4 |
| $OKT3^+$(%) | 55± 1.8 | 58± 1.6 | 58± 1.0 |
| Total T Cells* | 1135±82 | 1181±106 | 1105±87 |
| $OKT4^+$(%) | 37± 1.7 | 36± 1.7 | 31± 1.7 |
| $T_H$ Cells* | 681±50 | 725± 66 | 587±52 |
| $OKT8^+$(%) | 23± 1.3 | 23± 0.9 | 33± 2.8 |
| $T_S$ Cells* | 414±33 | 486± 56 | 616±54 |
| $T_H$:$T_S$ ratio | 1.7± 0.1 | 1.6± 0.1 | 1.1± 0.1 |

*±1 s.e.

**Table II** Mean values for age, total T cells and T lymphocyte subsets. Comparison of breast cancer patients, Stages I–III to Stage IV

| | Stage I–III | Stage IV |
|---|---|---|
| Age (yrs)* | 60± 3.0 | 64± 4.3 |
| $OKT3^+$(%) | 59± 2.0 | 58± 4.0 |
| Total T Cells* | 1224±114.0 | 919±106.0 |
| $OKT4^+$(%) | 35± 2.0 | 26± 2.0 |
| $T_H$ Cells* | 703± 59.0 | 404± 34.0 |
| $OKT8^+$(%) | 26± 1.4 | 45± 3.5 |
| $T_S$ Cells* | 548± 64.0 | 723± 85.0 |
| $T_H$:$T_S$ ratio | 1.4± 0.1 | 0.6± 0.06 |

*±1 s.e.

We wish to express our thanks to Professor J.M. Bridges for his helpful advice, Mr. C. Patterson for help with statistical analysis of data and Miss J. Cunningham for assistance in preparation of the manuscript.

### References

BOYUM, A. (1968). Separation of leukocytes from blood and bone marrow. *Scand. J. Clin. Lab. Invest.*, **21**, (Suppl. 97) 77.

CERVANTES, C., EXPOSITO, G., MORENO, L. & CARCIA, M.E. (1979). Immunological studies in breast cancer lymphoid populations. *Neoplasma*, **26**, 711.

CONESA, L.C.G., PASQUALINI, R.S., PUJATO, D. & LYNCH, M. (1979). Cellular immunity in early human breast carcinoma. *Cell. Mol. Biol.*, **24**, 355.

JERRELLS, T.R., DEAN, J.H. & HERBERMAN, R.B. (1978). Relationship between T lymphocyte levels and lymphoproliferative responses to mitogens and alloantigens in lung and breast cancer patients. *Int. J. Cancer*, **21**, 282.

KELLER, S.E., IOACHIM, H.L., PEARSE, R. & SILETTI, D.M. (1976). Decreased T lymphocytes in Patients with Mammary Cancer. *Am. J. Clin. Pathol.*, **65**, 445.

KUNG, P.C., GOLDSTEIN, G., REINHERZ, E.L. & SCHLOSSMAN, S.F. (1979). Monoclonal antibodies defining distinctive human T cell surface antigens. *Science*, **206**, 347.

MENCONI, E., BARZI, A., GRECO, M., CAPRINO, M.C., DE VECCHIS, L. & MUGGIA, G. (1979). Immunological profile of breast cancer patients in early or advanced disease. *Experientia*, **35**, 820.

OSBORNE, C.K., KNIGHT, W.A., YOCHMOWITZ, M.G. & McGUIRE, W.L. (1980). Modern approaches to the treatment of breast cancer. *Blood*, **56**, 745.

VAN WAUWE, J. & GOOSSENS, J. (1981). Monoclonal anti-human T-lymphocyte antibodies: Enumeration and characterization of T-cell subsets. *Immunology*, **42**, 157.

WHITTAKER, M.G. & CLARK, C.G. (1971). Depressed lymphocyte function in carcinoma of the breast. *Br. J. Surg.*, **58**, 717.

ZEMBALA, M., MYTAR, B., POPIELA, T. & ASHERSON, G.L. (1977). Depressed *in vitro* peripheral blood lymphocyte response to mitogens in cancer patients: The role of suppressor cells. *Int. J. Cancer*, **19**, 605.